# Technical Advances in the Measurement of Residual Disease in Acute Myeloid Leukemia

**DOI:** 10.3390/jcm6090087

**Published:** 2017-09-19

**Authors:** Gregory W. Roloff, Catherine Lai, Christopher S. Hourigan, Laura W. Dillon

**Affiliations:** Myeloid Malignances Section, Hematology Branch, National Heart, Lung, and Blood Institute, National Institutes of Health, Bethesda, MD 20892, USA; gregory.roloff@gmail.com (G.W.R.); catherine.lai@nih.gov (C.L.)

**Keywords:** acute myeloid leukemia, minimal residual disease, next-generation sequencing, error-corrected sequencing, droplet digital polymerase chain reaction, imaging

## Abstract

Outcomes for those diagnosed with acute myeloid leukemia (AML) remain poor. It has been widely established that persistent residual leukemic burden, often referred to as measurable or minimal residual disease (MRD), after induction therapy or at the time of hematopoietic stem cell transplant (HSCT) is highly predictive for adverse clinical outcomes and can be used to identify patients likely to experience clinically evident relapse. As a result of inherent genetic and molecular heterogeneity in AML, there is no uniform method or protocol for MRD measurement to encompass all cases. Several techniques focusing on identifying recurrent molecular and cytogenetic aberrations or leukemia-associated immunophenotypes have been described, each with their own strengths and weaknesses. Modern technologies enabling the digital quantification and tracking of individual DNA or RNA molecules, next-generation sequencing (NGS) platforms, and high-resolution imaging capabilities are among several new avenues under development to supplement or replace the current standard of flow cytometry. In this review, we outline emerging modalities positioned to enhance MRD detection and discuss factors surrounding their integration into clinical practice.

## 1. Introduction

Acute myeloid leukemia (AML) is a malignancy of blood-forming stem cells in which recurrent genetic mutations and chromosomal aberrations are associated with the clonal expansion of immature myeloid populations [1,2]. For those fit to undergo intensive induction therapy, treatment typically with a combination of anthracycline and cytarabine successfully induces cytomorphological complete remission (CR) in up to 70% of patients [3,4,5]. Despite this apparent initial success, the return of clinically evident disease (“relapse”), typically resulting in death, remains common [6]. Accordingly, predicting, detecting, and averting relapse after CR is a topic of active investigation.

For more than 50 years, CR in AML has been defined by the recovery of marrow function and peripheral blood counts after completion of chemotherapy and by a morphologic examination of the bone marrow revealing <5% myeloblasts [7]. With such a threshold, it is possible for a patient in cytomorphological CR to be harboring as many as 10^10^ leukemic cells in their bone marrow [8], suggesting that the definition of CR inadequately characterizes a vastly heterogeneous range of leukemic disease burden [9]. Current clinical decisions regarding the provision of consolidative therapy or hematopoietic stem cell transplant (HSCT) once CR is achieved depend on pretreatment correlates of the disease biology rather than direct assessment of the measurable disease remaining.

While the most recent response criteria for AML now does include a category of MRD-negative CR [10], there is no single standard technique for such sensitive detection; real-time quantitative PCR (qPCR) for overexpressed genes [11,12] or pathognomonic chromosomal translocations [13], fluorescence in situ hybridization (FISH) [14], and multiparameteric flow cytometry [15] are all possible detection strategies. Regardless of the MRD detection methodology used, it is widely appreciated that MRD positivity (MRD^+^) in cytomorphological CR portends a higher cumulative risk of subsequent clinically evident relapse. Similarly, patients with detectable MRD prior to HSCT are also at a high risk of post-transplant relapse [16]. Recent independent analyses from two different centers using two different MRD technologies found no significant difference in overall survival between patients in morphological CR but with MRD^+^ and patients not achieving CR prior to HSCT [17,18]. In this article, we detail exciting technical advances in molecular biology, next-generation sequencing (NGS), and imaging sciences and discuss the potential for using these new technologies to bring MRD detection into the 21st century. We close by outlining situations in which more precise detection and absolute quantification of MRD might guide clinical decision making. 

## 2. Next-Generation Sequencing

The most widely utilized MRD testing relies on quantitative or probe-based PCR approaches for the detection of chromosomal fusion sequences or mutation-specific sequences not typically observed in healthy individuals [19,20]. While these methodologies are well-established and sensitive, the molecular heterogeneity of AML limits the application of PCR-based MRD assays to only some molecular subsets. In AML cases harboring recurrent chromosomal fusions such as t(15;17)(q22;q21), t(8;21)(q22;q22.1), or inv(16)(p13.1q22), extensive efforts have led to the development of qPCR assays for tracking the disease status [19,21]. These assays serve only patients with favorable-risk disease, who represent only a minority of all AML cases [22,23]. Other PCR strategies targeting intermediate-risk patients with nucleophosmin (NPM1) insertion mutations, who comprise about 30% of all AML cases [10] and half of patients with normal cytogenetic profiles, first require identification of the insertion type and subsequent use of mutation-specific PCR primers for longitudinal tracking over time [20]. 

In 2008, AML was the first cancer genome to be fully characterized by NGS [24]. Since that time, numerous NGS projects have comprehensively depicted the clonal, heterogeneous biology of AML at presentation, relapse, or transformation from antecedent hematologic disease [1,25]. NGS offers several advantages over other technologies for MRD tracking. First, instead of cycling times allowing for the back-calculation of input nucleic acid, the operator is provided with an output at nucleotide resolution allowing for the identification of variants. Additionally, NGS enables the study of the entire mutational profile of a cancer genome, compared to a normal tissue counterpart. Massively parallel sequencing increases the throughput, allowing several samples to be multiplexed into a single experiment while testing for multiple targets. For these reasons, NGS represents an attractive platform for advancing MRD detection. 

### 2.1. DNA Sequencing

If NGS-based MRD testing is to have potential for clinical translation, its performance must match or exceed existing procedures for MRD detection. In 2012, Thol and colleagues demonstrated the feasibility of MRD detection by NGS. In 80 patients with fms related tyrosine kinase 3 internal tandem duplication (FLT3-ITD) or NPM1 mutations tracked at diagnosis and follow-up, DNA sequencing proved to be a reliable technique for the quantitative monitoring of NPM1 mutational load throughout the patient clinical course. This NGS-based tracking strategy revealed the emergence of dominant clonal populations and was found to be concordant with qPCR in 95% of cases [26]. Subsequent work by Kohlman et al. [27] examined the potential of amplicon deep sequencing of runt-related transcription factor 1 (RUNX1) mutations as an MRD marker in a prospective cohort of 814 patients over a 22 month period. Patients could be segregated into “good responder” or “bad responder” groups on the basis of median RUNX1 mutational burden at CR and followed distinct patterns of event-free survival and overall survival, respectively. 

Recent research by Getta and colleagues compared a 28 gene targeted DNA NGS panel to multiparametric flow cytometry (MFC) at diagnosis and before allogeneic HSCT [28]. NGS-based MRD was defined as having AML-associated alleles present at >5% variant allele frequency. The concordance between the two methodologies was 71%, and the patient samples testing positive for MRD by both techniques were associated with a higher risk of relapse, compared to either modality alone. This work demonstrated that a multi-gene NGS approach can augment MFC and provide actionable clinical information prior to transplantation.

Klco et al. have also demonstrated the prognostic capabilities of longitudinal NGS testing for MRD [29]. Importantly, after first establishing that single time-point genomic studies of adult AML samples at first presentation were not independently prognostic beyond established metrics (age, cytogenetics, and coding mutations in recurrently mutated genes), 50 individuals in CR on day 30 were studied at serial intervals using enhanced deep exon sequencing or a commercially available amplicon-based targeted platform of 264 recurrently mutated genes. Patients with clearance at day 30 of all relevant somatic mutations present at diagnosis had superior outcomes, compared to those with at least one persistent mutation.

### 2.2. Error-Corrected Sequencing

While these studies highlight the value and potential of NGS for tracking MRD, technical hurdles remain. Because many NGS library preparation procedures involve multiple rounds of PCR amplification, polymerase artifacts introduced during the procedure are contained within the output sequence. Massively parallel sequencing itself also carries an inherent error rate. For Illumina instruments, this generally ranges from 0.05% to 1.0% of calls [30,31] and is influenced by the read length and bioinformatics algorithms used during analysis [32]. In clinical research situations in which samples are genetically heterogeneous, such discrepancies may cloud the detection of true mutants present at a low allele frequency, and thus highly sensitive detection of rare subclones contributing to MRD in AML represents an obstacle to the clinical application of NGS. Additionally, varying efficiencies in the PCR amplification step can lead to bias in the representation of various sequences in the final library, making comparisons of the copy number challenging [33]. 

**Figure 1:**
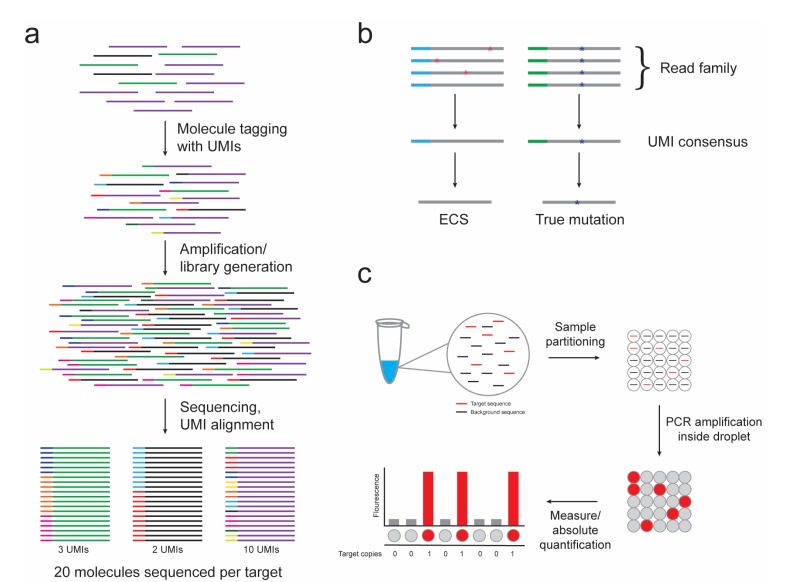
The use of unique molecular indexes (UMIs) in next-generation sequencing (NGS) libraries and droplet digital PCR (ddPCR) provide technical advances for measurable residual disease (MRD) detection. (**a**) Molecules consisting of three different nucleic acid targets (purple, green, and black lines) are individually tagged with UMIs. The UMI tags are maintained throughout amplification and library generation and are used to count the number of original target molecules present in the sample despite PCR amplification bias. (**b**) Alignment of read families (grey line) sorted by UMIs (blue or green line) allows for the discrimination of true mutations (blue asterisk) from sporadic errors introduced during PCR or sequencing procedures (pink asterisk). (**c**) A single PCR reaction is partitioned by ddPCR into thousands to millions of water–oil droplets for which no more than a single target sequence (red line) is present per droplet. PCR amplification is performed within the droplets and the fluorescent signal is measured as either positive or negative, allowing for absolute target copy number quantification.

To remediate these obstacles, the use of random barcodes or unique molecular indexes (UMIs) has been introduced to various NGS methods [34,35,36,37,38,39,40]. UMIs can be designed as a string of completely random, partially degenerate, or defined nucleotides, depending on the number of template molecules to be identified. Sample preparation involves the introduction of a UMI to each target molecule prior to PCR amplification. PCR progeny (read family) of a single target molecule can be identified by aligning common UMIs (Figure 1a). This method allows for a digital measurement of each target molecule, thus removing bias introduced by PCR amplification and allowing for the accurate quantification and comparison of targets. 

Additionally, bioinformatics methods that utilize UMIs to discriminate rare variants from protocol artifacts, referred to as “error-corrected sequencing” (ECS), have been developed (Figure 1b). Mutations in the template molecule are expected to be propagated into each daughter molecule of a common UMI read family, while variations due to procedural errors will be sporadically dispersed. Some groups have set thresholds (i.e., 90%) for variant calls, above which the members of a read family must be identical to be considered a true mutant allele [34]. Inconsistencies below this level (PCR or base-calling errors) are adjusted to yield an error-corrected consensus sequence. Other strategies rely on analyzing relationships in minor base differences in similar UMI sequences and devising algorithms to identify and amend errors [41]. Recently, Young and colleagues reported the first proof-of-principle experiment of ECS in clinical samples from AML patients [42]. Using 16 bp oligonucleotide adapters, a leukemia-specific variant allele fraction of 1:10,000 was consistently able to produce a digital discrimination of tumor DNA within a heterogeneous input sample. ECS-mediated detection of leukemia-associated mutations in banked blood and bone marrow samples allowed for the identification of subclones below the traditional threshold of detection for standard NGS. 

Another highly accurate ECS approach, duplex sequencing [43], uses ligation of double-stranded UMIs to guide error identification. Because two strands of barcoded DNA can be assessed, true mutants can be disentangled from early PCR or sequencing errors that are carried forward only from one strand. Duplex sequencing has been demonstrated to have the lowest theoretical error rate of existing technologies, as less than one artifact mutation per billion nucleotides is sequenced. 

### 2.3. RNA Sequencing 

While NGS has been employed in AML patients to detect MRD-relevant sequence mutations, monitoring the aberrant expression of wild-type genes also serves as an important target for MRD testing [11,44]. To this end, an RNA-sequencing (RNA-seq) approach presents a promising new methodology for MRD testing. Historically, gene expression was determined by microarrays, which provide robust, reliable data in a relatively high-throughput manner. However, the dynamic range of microarrays is limited, making them unsuitable for the detection of AML MRD. Although qPCR has been used for gene expression-based MRD testing [11,19,21,44,45], the inability to detect multiple targets within a single sample has limited widespread adoption. In recent years, RNA-seq has emerged as an attractive platform because of its ability to overcome these problems while identifying novel transcripts, transcriptional modifications, fusions, and other structural variations at a single-nucleotide resolution. Additionally, an RNA-seq-based approach has the advantage over a DNA-seq-based approach in that it: (1) allows for simultaneous tracking of mutations and changes in transcript expression, and (2) could provide a deeper level of detection if the expression level of the transcript of interest is greater than the genomic copy number per cell. However, the use of RNA requires great care to be taken with the pre-analytical workflow to prevent degradation, although standard operating procedures for this are already common in most clinical molecular pathology laboratories. In addition, RNA sequencing may require supplementation by DNA-based assays for targets in which mutated alleles have no or low detectable transcripts.

RNA-seq has already been successfully employed to reveal gene fusions in numerous AML patients with normal cytogenetics [46,47,48]. In the future, multi-gene targeted RNA-seq panels could greatly expand the repertoire of AML MRD-tracking NGS applications. The addition of UMIs and ECS approaches to this technique has the potential to deeply detect and quantify MRD across a wide variety of mutation types in a single reaction. 

## 3. Digital PCR

PCR allows for both the qualitative and quantitative detection of genetic alterations in cancer. The molecular detection of MRD was significantly advanced through the development of qPCR assays [19,21], which quantitatively measure AML-associated mutations and gene expression changes using secondary fluorescence measurements. While advancing the field greatly, qPCR assays require the use of standard curves and rigorously controlled standards for accurate detection. Additionally, various parameters, such as amplification bias, can influence the output measurement and lead to inaccurate quantification. MRD detection in the clinical research setting requires the ability to confidently detect disease-relevant mutations present at very low frequencies. Digital PCR may represent a solution to many or all of these issues.

The concept of digital PCR was first introduced in the 1990s, when investigators devised dilution strategies to partition a single PCR reaction into numerous reactions, allowing for the amplification of individual template molecules [49,50]. By isolating no more than an individual DNA molecule per partition, the PCR reaction is converted from analog to digital in nature, and the output signal is either positive or negative, allowing for absolute quantification. Technical advances now enable template molecules to be fractionated into thousands to millions of water–oil droplets for which PCR amplification of the target occurs within each droplet, referred to as droplet digital PCR (ddPCR) (Figure 1c) [51]. Alternatively, chip-based systems forego droplet emulsions and distribute PCR reactions into nanoliter fluidics platforms [52,53]. There are now multiple commercial platforms available. 

Digital PCR represents a promising technique for the sensitive, specific, and personalized molecular characterization of patient samples across multiple cancer types [54]. Given the diversity of somatic mutations and chromosomal aberrations involved in the pathogenesis of AML [55], digital PCR is an attractive target to deeply probe for patient-specific MRD markers. Because digital PCR may use probe-based chemistry [56], hotspot regions corresponding to a wild-type sequence, mutations, or structural cytogenetic changes can be detected using specifically designed assays based on a patient’s molecular characteristics.

Compared to NGS, digital PCR takes a faster time to find each result (after initial assay development) and is currently more sensitive with the detection of mutant alleles to frequencies as low as 0.001% possible with commercially available platforms [57]. Digital PCR also has a lower error rate and does not require the use of complex bioinformatics pipelines, both of which hinder the adaptation of NGS techniques in a clinical setting. Despite the many advantages of digital PCR, there are also limitations. Like qPCR, digital PCR requires that customized assays be developed for each individual target, making it most useful as a follow-up after a suitable target for monitoring has been identified. Additionally, while multiplexing is possible, typically only a few targets can be monitored simultaneously within each sample. 

While data for the application of digital PCR in AML is still emerging, early results demonstrating the feasibility of such an approach are exciting. Brambati and colleagues developed ddPCR assays for DNA (cytosine-5)-methyltransferase 3A (DNMT3A), isocitrate dehydrogenase 1 (IDH1), and isocitrate dehydrogenase 2 (IDH2) [58], three genes that are commonly mutated in AML and that have been shown to occur early in the course of leukemogenesis [25,58]. Conventional NGS approaches were first employed to screen for relevant mutations in a cohort of 89 AML patients prior to HSCT. Mutant-specific ddPCR primers and probes were generated and utilized in the pre- and post-transplant setting for serial tracking in 30 patients with mutations in DNMT3A, IDH1, or IDH2. In all cases, samples positive for the mutations-of-interest pre-transplant by NGS were also detectable by ddPCR. Furthermore, all 14 cases with DNMT3A mutants and all but one case with IDH1/2 mutations pre-HSCT had recurrent mutations detectable post-transplant. 

Mencia-Trinchant et al. recently utilized ddPCR to track MRD by focusing on NPM1 mutants, which represent 30% of all AML cases and more than half of those with normal cytogenetics [59]. Approximately 95% of NPM1 mutations in AML patients consist of a four-nucleotide insertion in exon 12, which ordinarily requires specific assays and standards for each possible insertion for quantification by qPCR [20]. Mencia-Trinchant et al. circumvented this problem by using a multiplexed pool of primers to account for mutant-specific sequence variation, thus capturing nearly all NPM1 mutant types. Proof-of-principle experiments revealed the detection of rising NPM1 mutants in the peripheral blood of three patients, each with a unique mutation type. Relapse eventually occurred in all three patients, demonstrating the predictive power of serial mutation-based MRD profiling and the multiplex power of ddPCR. 

## 4. Novel Imaging Technologies

Bone marrow examination is currently the “gold standard” for accurate measurement of residual leukemic burden in AML patients. Cytomorphologic examination of the bone marrow is typically carried out at diagnosis (baseline), at the completion of induction therapy, and if applicable, at relapse. Mid-treatment biopsies may be performed, but they are of controversial utility. With the improvement of peripheral blood monitoring techniques, in combination with better imaging modalities, it may be possible to create a new standard for evaluating the response to treatment by minimizing the need for painful biopsies [60]. As a result of the cost and time needed to complete clinical trials, there are a lack of studies exploring imaging in AML. Because rigorous studies exploring imaging as a potential modality for MRD assessment are lacking, imaging is not currently used in the evaluation of AML patients. Nevertheless, as technologies evolve, imaging may serve to augment our current capabilities to predict treatment response or enhance prognostication.

For example, positron emission tomography (PET) has been investigated for the ability to detect latent disease burden in AML patients. PET is a functional imaging technique used to evaluate metabolic processes. There are two types of PET scans that have been used in AML patients. In fludeoxyglucose (FDG) PET, a biologically active analogue of glucose is used as a tracer. FDG PET sensitively measures glucose uptake as a function of metabolic activity. However, in the majority of cases, FDG PET is not specific for distinguishing anti-tumor inflammatory response, infection, or glucose uptake secondary to tumor progression. Interestingly, FDG PET has shown efficacy in visualizing extramedullary disease (EMD). While AML is a disease of the bone marrow and can be diagnosed and followed with biopsies, EMD can present in any organ and is currently thought to be present in approximately 25% of new diagnoses [61]. In a small study of 10 patients, FDG PET was able to detect known EMD in 90% of the patients and additional EMD in 60% [62]. In another study of 26 patients evaluated with newly diagnosed AML, FDG PET found 65% of the patients to have EMD, compared to 31% found by clinical exam [63]. While the sensitivity seen in FDG PET can be as high as 93%, the specificity is only 71%, creating a number of false positive cases [64]. While the utility of FDG PET is unknown and sensitivity must be balanced with specificity and accuracy, there may be a role for this imaging modality as an adjunct at diagnosis for patients with EMD AML to determine the extent of the disease and at the end of treatment to document response [65].

Alternatively, [F18] 3’-deoxy-3’-fluorothymidine (^18^F-FLT) PET may be more suitable for the evaluation of AML patients, given that ^18^F-FLT is a thymidine analog that is resistant to in vivo degradation and accumulates in proliferating tissues, including rapidly dividing hematopoietic stem cells in the bone marrow [66]. In AML patients, ^18^F-FLT PET shows a higher rate of bio-distribution in the bone marrow, spleen, and EMD compared to normal healthy controls [67]. Used as an early assessment of treatment response in eight newly diagnosed AML patients treated with induction chemotherapy, ^18^F-FLT PET imaging was carried out between two and six days after the start of treatment. Patients with a CR showed a standardized uptake value (SUV) of <2, while patients with resistant disease (RD) displayed a SUV of >2. SUV_mean_ and SUV_max_ were also significantly lower in patients with CR compared to RD, and normal controls had SUVs similar to that of patients in CR [68]. Although the patient sample size in this study was too small to make any generalizations or conclusions, it addresses an interesting question of applying imaging as an early response assessment tool. For patients who do not respond to initial therapy, it may be worth changing treatment early to avoid unnecessary toxicity from an unsuccessful regimen. Currently, ECOG-ACRIN Cancer Research Group is conducting a phase 2 study of ^18^F-FLT PET/computed tomography (CT) at the time of the nadir bone marrow (days 10–17) in newly diagnosed AML patients being treated with standard induction chemotherapy (NCT02392429). 

Further studies must be conducted before imaging technology can be incorporated into standard assessments for AML monitoring. At present, it is unknown which imaging modality would be used, if any, and when the ideal time to insert into practice would be, either before, during, or after treatment. In the era of increasingly sensitive techniques for the detection of MRD, it will be important to consider the timing, combination, and sequence of methods used for the diagnosis and follow-up of AML patients. Ultimately, if imaging is to have utility in AML, it is likely to be in triggering a more sensitive assessment by bone marrow examination in a patient testing negative by peripheral blood assays alone and/or for the detection of EMD.

## 5. Conclusions

It is increasingly appreciated that MRD testing adds to prognostication in AML beyond established metrics such as patient age and cytogenetics [60,69,70]. Flow cytometry is the standard method for the measurement of residual disease in most subtypes of AML. Where available, however (currently only for “favorable risk” subtypes, e.g., APL, core binding factor leukemia, and NPM1-mutated AML), molecular methods are preferred because of their high reproducibility and inter-center standardization. While excellent MRD qPCR testing exists for example in the case of acute promyelocytic leukemia, as a result of improvements in therapy outcomes, it can be argued that these patients with low risks of relapse need MRD testing least [71]. There is a need to develop more widely applicable molecular- or genomic-based assays capable of being tested in clinical trials to determine the value of MRD monitoring for all other subtypes of AML.

The MRD-tracking methodologies described above will likely augment our armamentarium by offering several unique advantages beyond what current techniques provide. As discussed for cases with NPM1 mutations, NGS mitigates the need for DNA sequencing-based mutant identification prior to longitudinal tracking with specific PCR primers and standards. Additionally, multiplexed primer designs can be formulated to capture mutations, fusions, and expression changes. Unlike traditional PCR with limitations surrounding amplification bias and inconsistencies with assay efficiency, ddPCR provides a direct measurement of target molecules rather than an estimate of target sequences based on a relationship to cycling time. Finally, novel imaging modalities have the potential to monitor extramedullary disease not detectable by blood or bone marrow assessment while also potentially informing the timing for invasive bone marrow biopsies. 

Several factors, both technical and systemic, currently pose barriers to the integration of such technologies for routine MRD monitoring. The inherent sensitivity and error rates between commercially available NGS platforms can vary markedly, especially for low-frequency variants, and will likely require synchronization [72]. Additionally, inconsistencies among base-calling analysis algorithms must be minimized, and several techniques have been developed to aid in this task [73]. However, the most important of these obstacles is a understandable reluctance by physicians to interpret a MRD^+^ test result status alone as a clinically actionable predictor of relapse [74]. While decision-making for the majority of AML cases continues to be guided by CR criteria devised in 1956, the expanding body of evidence demonstrating the value of MRD testing, along with rapidly advancing technologies, should inspire hope for the ample opportunity that exists to strengthen the information available to hematologists during decision-making, and therefore ultimately improve patient outcomes. The completion of several large, randomized, multi-institutional clinical trials will likely contribute powerful evidence for the transition towards the MRD testing-based management of AML. While initially, clinical testing will most commonly be by flow cytometry, the future for molecular detection of AML MRD looks bright.
